# A Straightforward Access to New Families of Lipophilic Polyphenols by Using Lipolytic Bacteria

**DOI:** 10.1371/journal.pone.0166561

**Published:** 2016-11-17

**Authors:** Leyre Sánchez-Barrionuevo, Alejandro González-Benjumea, Almudena Escobar-Niño, María Teresa García, Óscar López, Inés Maya, José G. Fernández-Bolaños, David Cánovas, Encarnación Mellado

**Affiliations:** 1 Department of Genetics, Faculty of Biology, University of Seville, Seville, Spain; 2 Department of Microbiology and Parasitology, Faculty of Pharmacy, University of Seville, Seville, Spain; 3 Department of Organic Chemistry, Faculty of Chemistry, University of Seville, Seville, Spain; Wageningen Universiteit, NETHERLANDS

## Abstract

The chemical synthesis of new lipophilic polyphenols with improved properties presents technical difficulties. Here we describe the selection, isolation and identification of lipolytic bacteria from food-processing industrial wastes, and their use for tailoring a new set of compounds with great interest in the food industry. These bacteria were employed to produce lipolytic supernatants, which were applied without further purification as biocatalysts in the chemoselective and regioselective synthesis of lipophilic partially acetylated phenolic compounds derived from olive polyphenols. The chemoselectivity of polyphenols acylation/deacylation was analyzed, revealing the preference of the lipases for phenolic hydroxyl groups and phenolic esters. In addition, the alcoholysis of peracetylated 3,4-dihydroxyphenylglycol resulted in a series of lipophilic 2-alkoxy-2-(3,4-dihydroxyphenyl)ethyl acetate through an unexpected lipase-mediated etherification at the benzylic position. These new compounds are more lipophilic and retained their antioxidant properties. This approach can provide access to unprecedented derivatives of 3,4-dihydroxyphenylglycol with improved properties.

## Introduction

The beneficial effects of the Mediterranean diet are partly due to its high content in antioxidant compounds [1]. In particular, the polyphenols present in the virgin olive oil display a strong antioxidant activity *in vitro* [2] and *in vivo* [3,4], which has impelled a growing interest in these compounds, especially those that can be obtained from by-products of the food industry [5]. Several epidemiological studies have shown the beneficial health effects arising from consumption of foods rich in antioxidants, preventing the damage caused by prolonged oxidative stress in certain biomolecules (nucleic acids, lipids, proteins), which is associated with an increased risk of chronic diseases [6]. The acylated polyphenols display improved properties as functional ingredients compared to the natural polyphenols [7] as they are lipophilic antioxidants with improved resistance against metabolic degradation, and they can also be incorporated into lipid food matrices such as fats and oils, processed foods, and margarines [8,9].

Protection and deprotection of functional groups are commonly employed in organic chemistry to carry out the synthesis of partially acylated derivatives [10]. However, despite of the many advances in organic synthesis during the last decades, the conventional chemical synthesis of mono acylated derivatives of polyphenols presents serious difficulties due to the high density of very similar functional groups, which requires extensive protection and deprotection sequences [10]. Thus, although some chemical approaches have been developed for accessing partially acylated polyphenols in a chemoselective way [11–13], most of such procedures are pure chemical synthesis, and involve the use of hazardous reagents or non-green conditions. As an alternative, the biotech industries have traditionally produced compounds of commercial interest by microbial fermentation, or by fermentation followed by subsequent chemical modification to improve specific properties, such as activity, solubility, absorption, pharmacokinetics or stability. In this context, enzymes and microorganisms have been efficiently used as biocatalysts in chemo-, regio- and stereoselective synthesis of bioactive compounds.

We reason that an enzyme-catalyzed approach could offer a viable alternative to traditional fermentation procedures and conventional chemical methods for the chemo- and regioselective synthesis of these new lipophilic compounds [14]. In this work, special attention has been focused on hydroxytyrosol (HT) and 3,4-dihydroxyphenylglycol (DHPG), which predominate in leaves and fruits of olive trees (*Olea europea*), either free or as acyl derivatives, and display antioxidant activity [15]. We have developed a novel enzymatic method to obtain targeted mono- or di-acylated derivatives of the polyphenols.

## Materials and Methods

### Site description and sample collection

The bacterial strains used in this study were isolated from locations in the provinces of Badajoz and Huelva (Spain) in 2010. Samples HR11 and HR12 contained semisolid fats from a meat curing factory (38.151216°N, -6.684258°E). Sample HR11 was obtained by collecting the dripping fat from the floor of the factory premises. Sample HR12 was obtained from a tank containing fat leftovers. Sample HR21 consisted of the fish dust that results after cutting fish into pieces before canning in a fish canning factory (37.20994°N, -7.26167°E). All samples were collected in 50 ml sterile plastic tubes and stored at 4°C until use.

### Screening to detect lipolytic microorganisms (hydrolysis)

Fish sawdust samples (7.28 g) were suspended in 25 ml of sterile saline solution (NaCl 0.85% w/v). In the case of the sample from cured meat oil, 5 ml of each sample of fat were suspended in 20 ml of sterile saline solution (NaCl 0.85% w/v). Screening for lipolytic microorganisms was performed as previously described [16].

### Transesterification assay of lipolytic activity

Transesterification activity of lipase was tested by a colorimetric method with minor modifications [16]. This method is based on the release of the yellow-colored compound *p*-nitrophenol (*p*-NP) after the transesterification of *p*-nitrophenyl palmitate (*p*-NPP; Sigma-Aldrich) with ethanol, and the subsequent detection by using UV-Vis spectrophotometry. Strains producing the maximum lipase activity were selected for further studies.

### Optimization of bacterial growth conditions and lipase production

In order to optimize the production of the bacterial lipases, the strains were grown in two different media (PYB or LB) in the presence or in the absence of 2% tributyrin (PYBT or LBT media). PYB medium contains 1% (w/v) peptone, 0.5% (w/v) yeast extract, 0.1% (w/v) K2HPO4, 0.02% (w/v) MgSO4 ·7H2O. pH was adjusted to 7.5. LB medium (1% (w/v) tryptone, 0.5% (w/v) yeast extract, 0.5% (w/v) NaCl) was supplemented with 2% (w/v) glucose. Bacteria were grown in 500 ml Erlenmeyer flasks containing 100 ml of medium. Growth was monitored by measuring the absorbance at 600 nm (OD_600_) in a Beckman DU640 spectrophotometer. The lipase activity was assayed employing the *p*-NPP method described at 37°C.

### Purification of the supernatants

The cell-free supernatant was obtained by centrifugation of bacterial cultures at 4,500 rpm for 5 min at 4°C. These supernatants were concentrated in dialysis bags (12 kDa, Sigma) against polyethylene glycol (8 kDa) at 4°C overnight, and then, they were dialyzed in 0.05 M potassium phosphate buffer (pH 7.6). Finally, they were freeze-dried. The dry supernatants were employed as enzymatic cocktails for the lipase assays.

### Isolation of DNA and 16S rRNA gene sequence analysis

Bacterial DNAs were isolated and used for the amplification of the 16S rRNA by PCR using the universal primers 16F27 (5′-AGAGTTTGATCMTGGCTCAG-3′) and 16R1488 (5′-CGGTTACCTTGTTAGGACTTCACC-3′) as previously reported [16]. 16S rRNA sequences corresponding to positions 53 to 667 of the 16S rRNA gene from *Escherichia coli* were obtained and analyzed as previously described [16].

### Nucleotide sequence accession numbers

The nucleotide sequences were deposited under accession numbers KP212109 to KP212128 in the GenBank database.

### General methods for the chemoenzymatic syntheses of acylated polyphenols

NMR spectra were recorded at 25°C on a Bruker Avance 300 spectrometer, on a Bruker Avance III 500 MHz, and on a Bruker Avance III 700 MHz instruments equipped with a cryogenically cooled 5 mm TCI gradient probe. Chemical shifts are reported in ppm (δ) and spectra were referenced to the residual protonated solvent (3.31 and 49.0 ppm for CD_3_OD, 7.26 and 77.2 ppm for CDCl_3_, 2.05 and 29.8 for (CD_3_)_2_CO, for ^1^H and ^13^C NMR, respectively). Coupling constants (*J*) are expressed in Hertz (Hz). The assignments of ^1^H and ^13^C signals were confirmed by 1D and 2D NMR experiments (COSY, HSQC, HMBC). High resolution mass spectra were obtained by LSIM using a Hewlett Packard 5989 A spectrometer coupled to a Hewlett Packard 5990 II gas chromatographer and a Micromass AutoSpec-Q spectrometer with a resolution of 1,000 or 10,000 (10% valley definition); a cesium gun, 1-thioglicerol as matrix and NaI as additive were used. Column chromatography was performed using Merck silica gel 60 (230–400 mesh). TLCs were performed on silica-coated aluminum sheets from Merck (silica gel 60 F254) using mixtures of CH_2_Cl_2_−MeOH and EtOAc−hexane as eluants; spots were visualized by UV light and by staining with vanillin/H_2_SO_4_ in EtOH (1.5 g of vanillin in 100 mL of 95% EtOH/conc H_2_SO_4_ 100:1).

### Lipase-mediated acetylations of polyphenols 1, 4 and 8

The *O*-acetylations of the phenolic compounds were performed using the four lipolytic bacterial extracts, in a substrate-lipase extract 1:1 ratio in weight (40 mg). Isopropenyl acetate was used as solvent and acylating agent, 40 equiv (0.52–0.63 mL). The mixture was stirred at 40°C for 24 h in darkness. In the case of glycol derivative **4**, DMF was used as co-solvent because of solubility problems, isopropenyl acetate-DMF 1:1 in volume (1.0 mL).

### Lipase-mediated deacylation of compounds 2, 5 and 9

For the deacylation reactions of peracetylated polyphenols, to a solution of **2**, **5** and **9** (50 mg) in a primary linear aliphatic alcohol (MeOH, EtOH, propan-1-ol or butan-1-ol) was added silica gel and the lipase extract (from *Bacillus* sp. HR21-6 or *Terribacillus* sp. 2B122), in a substrate-alcohol-lipase extract-silica gel 1:50:2:2 ratio in weight. The mixture was heated at 60°C for 24–168 h until total conversion (in the case of *Terribacillus* sp. 2B122 lipase a second addition of lipase extract was needed when the reaction rate is markedly reduced). Finally, the solvent was evaporated and the residue was purified by column chromatography to give **3**, **6a-d** and **10**, respectively, using EtOAc-hexane or CH_2_Cl_2_-MeOH gradients as eluants (see S1 Supplementary Experimental Procedures).

### Hydroxytyrosyl acetate (3) from compound 2

Eluted with EtOAc-hexane 1:1 gave **3** (90% with *Bacillus* sp. HR21-6 or *Terribacillus* sp. 2B122) as a colorless syrup. Spectroscopic data for **3** are in agreement with those reported in literature [17].

### Deacetylation reactions of compounds 6a-d

To a solution of **6** (40 mg) in dry MeOH (1 mL) were added Cs_2_CO_3_ (2 equiv.) and sodium ascorbate (1 equiv.). The mixture was stirred in the darkness and in the presence of Ar at room temperature for 2–3 h. Then, the product was purified without evaporation of the solvent by column chromatography to give **7a-d**, using CH_2_Cl_2_−MeOH, CH_2_Cl_2_−EtOH, and EtOAc−hexane gradients as eluants (see S1 Supplementary Experimental Procedures).

### 4-(Methoxymethyl)benzene-1,2-diol (10) from compound 9

Eluted with EtOAc-hexane 1:1 gave **10** (quant. with *Bacillus* sp. HR21-6) as a colorless syrup. Spectroscopic data for **10** are in agreement with those reported in literature [18]. Supplementary experimental data procedures include additional information for the compounds **6a-d** and **7a-d**.

### DPPH radical scavenging activity

The antiradical activity of **6a-d** and **7a-d** has been evaluated by the DPPH method [19,20].

## Results

### Lipase-mediated *O*-deacylation of peracetylated HT

In order to obtain lipophilic phenolic esters it is essential to achieve the esterification of primary alcoholic groups without affecting the catechol moiety, which is known to be essential for the antioxidant effects. This process requires a chemoselective procedure.

**Fig 1:**
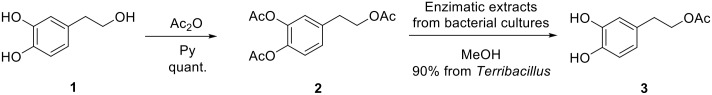
Regioselective deacetylation of peracetylated HT (2) catalyzed by the supernatants from bacterial cultures.

In a previous work we isolated bacteria capable of performing transesterification reactions in a regioselective fashion [16]. We reasoned that those bacterial isolates could also be used for the regioselective acylation/deacylation of polyphenols. For an initial analysis, HT (**1**) was firstly peracetylated with acetic anhydride in pyridine to obtain **2**, as previously described [21]. The deacetylation of the peracetylated HT was efficiently achieved using the supernatants of a bacterial culture obtained from our best performing strain (*Terribacillus* sp. 2B122) and an aliphatic alcohol as solvent (MeOH). The transesterification reaction was completely chemoselective in the phenolic positions, and gave a monoacetylated derivative in the aliphatic position (compound **3,** [22] as depicted in Fig 1, a natural compound present in extra virgin olive oil [23]).

### Screening for additional microorganisms showing lipolytic activity

In the previous reaction, the deacetylation was completely selective towards the phenolic esters, but we did not observe any selectivity towards the aliphatic ester moieties with this or other strains previously isolated. Therefore, we designed a new screening aiming at finding microorganisms capable of transesterification the aliphatic ester selectively. We reasoned that food industries involved in the processing of foods with high fat content would provide the best environment. Since a vegetable oil-rich location was screened previously, a fish and a meat industries located near Isla Cristina (Huelva, Spain) and Higuera la Real (Badajoz, Spain), respectively, were selected for sampling this time. After a first round of selection over 4,000 colonies (fungi and bacteria) were isolated from fish sawdust and from cured meat fat. Out of those, 459 were considered as positive (S1 Table). Interestingly, no lipolytic bacterial isolates were selected from the sample HR12 obtained from a meat curing factory, where the number of microorganisms obtained was 378. The lipolytic bacteria were grown in a non-selective medium (i.e. in the absence of tributyrin), and the lipolytic activity was then confirmed on LB supplemented with 0.5% tributyrin. These samples were transferred to the secondary screening.

### Screening for microorganisms capable of performing transesterification reactions among the selected lipolytic strains

The second step of the screening was performed using freeze-dried supernatants of the 66 bacterial isolates selected after the first step of the screening. The supernatants were assayed for transesterification activity by quantification of the yellow-colored *p*-nitrophenol (*p*-NP) that is released after the transesterification of *p*-nitrophenyl palmitate (*p*-NPP) with ethanol to give acetyl-palmitate ester and *p*-NP. We performed control assays as previously described [16]. The maximum value obtained in these control reactions was 0.119 and it was set as the threshold. Out of the 66 lipolytic bacterial strains in this second screening, 20 supernatants produced absorbance values higher than the cut-off of 0.119 (S2 Table). Most of the supernatants from the bacterial isolates displayed values between the cut-off and 0.300 and only very few were capable of displaying higher activities (4 isolates).

### Phylogenetic classification of the selected lipolytic bacteria

**Fig 2:**
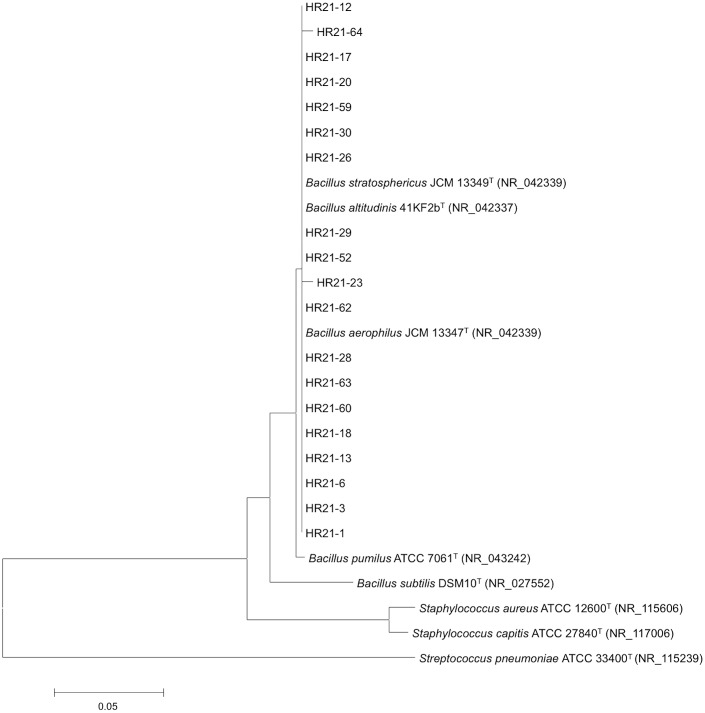
Evolutionary relationships of the selected strains. The phylogenetic tree shows the position of the isolates displaying lipolytic activity with respect to other type strains of genus *Bacillus* and an external bacterial group. The distances were calculated using Maximum Composite Likelihood. 16S rRNA gene sequences from the isolates correspond to 614 bps.

Phylogenetic studies were conducted using partial 16S rRNA sequences with the aim of identifying the bacterial isolates. Significantly, all strains isolated belong to the Gram-positive bacteria and were closely related to members of the genus *Bacillus* exhibiting similarity values ranging from 98% to 100%. The phylogenetic reconstruction carried out with different methods was consistent and suggested that all the strains (HR21-1, HR21-3, HR21-6, HR21-12, HR21-13, HR21-17, HR21-18, HR21-20, HR21-23, HR21-26, HR21-28, HR21-29, HR21-30, HR21-52, HR21-59, HR21-60, HR21-62, HR21-63, HR11-64 and HR11-65) clustered together, exhibiting a high similarity (98% to 100%) to the 16S rRNA sequences of *Bacillus aerophilus*, *Bacillus stratosphericus* and *Bacillus altitudinis* (Fig 2).

### *O*-Deacylation of peracetylated HT by *Bacillus* lipases

Since most of the isolates belonged to the *Bacillus* genus and were closely related, we only tested two random isolates for deacylation of HT. However, the results were similar to those obtained previously in Fig 1. Since the phylogenetic reconstruction using partial 16S rRNA sequences did not clearly differentiate between several species belonging to the genus *Bacillus*, we selected one of those two isolates for further studies and sequenced the complete 16S RNA. After further analysis, HR21-6 appeared to cluster together with *B*. *pumilus* and not with *B*. *stratosphericus*, *B*. *aerophilus* or *B*. *altitudinis* (S1 Fig).

### Optimization of the bacterial growth conditions for the production of lipases

The study was continued with four isolates belonging to different genera for tests in acylation and deacylation reactions of natural polyphenols. Three of the strains were previously isolated by our research group (the Gram negative *Pseudomonas* sp. 2B120 and *Enterobacter* sp. 1B89, and the Gram positive *Terribacillus* sp. 2B122) [16] and the fourth strain, *Bacillus* HR21-6, was isolated during this work (as described above).

**Fig 3:**
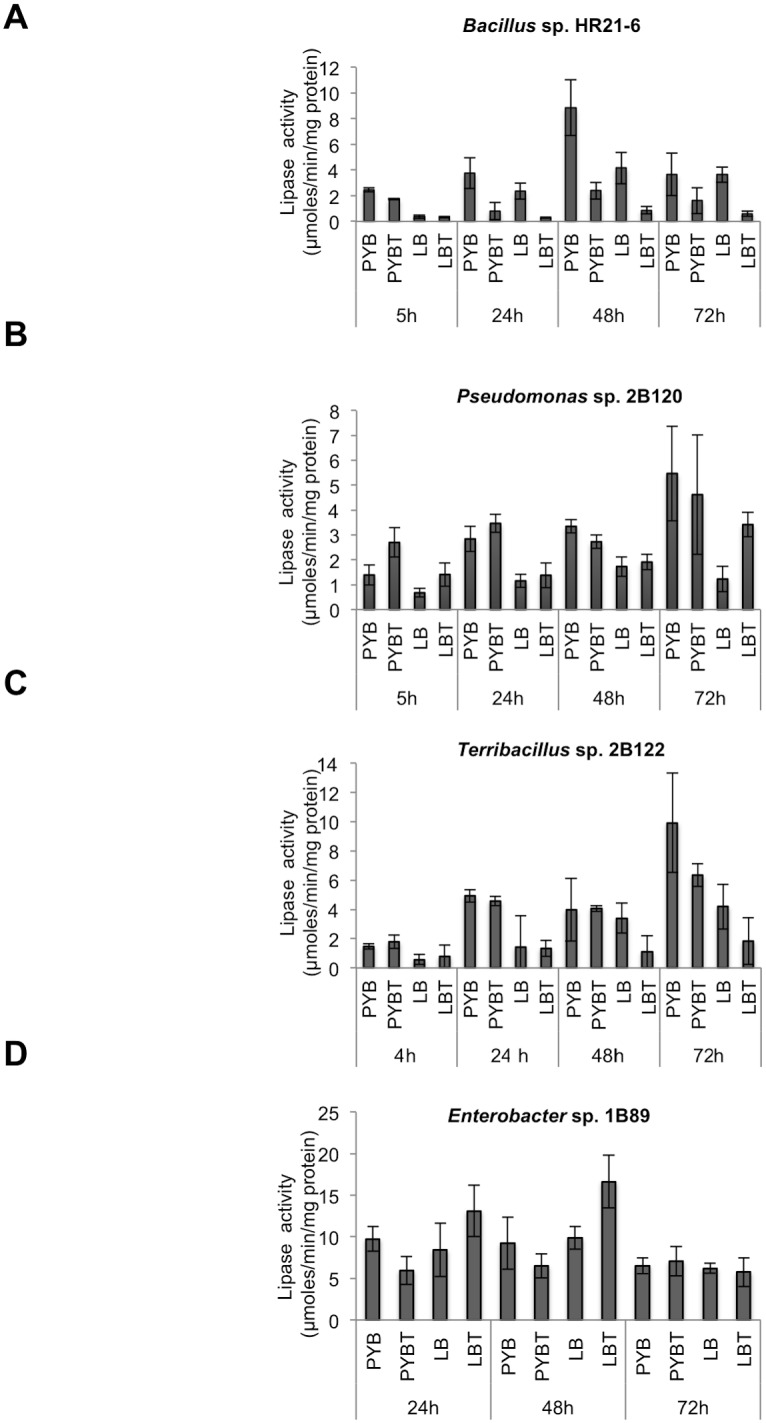
Optimization of growth conditions of the selected strains for the detection of lipase activity. To determine de lipase activity the bacterial strains, *Bacillus* sp. HR21-6 (A), *Pseudomonas* sp. 2B120 (B), *Terribacillus* sp. 2B122 (C) and *Enterobacter* sp. 1B89 (D) were grown in PYB or LB media with (PYBT or LBT) or without (PYB or LB) tributyrin for the indicated times. Lipase activity in the supernatants was quantified by using the *p*-NPP method. Data shown are the average of at least 3 independent experiments and the standard deviation of the mean.

An important factor in this study was to define the optimal conditions for the production of the lipase-rich supernatants. Two parameters were selected for designing the optimization variables: medium and incubation time. At this stage it was not known whether the lipases responsible for the transesterification activity observed were induced by lipidic substances or constitutively expressed. Therefore, we also added the lipidic substrate tributyrin (previously used for the screening) to the media. The effect of the different culture media in lipase activity at various time intervals is shown in Fig 3. In general, the activity of the supernatants of *Bacillus* sp. HR21-6 was higher when the microorganism was grown in PYB than in LB medium. The maximum activity was obtained at 48 h in PYB medium, which corresponded with the late stationary phase. In LB medium, the maximum activity was reached after 24 h of growth (Fig 3A). In both cases, addition of tributyrin produced a decrease in the lipase activity.

For *Pseudomonas* sp. 2B120 the lipase production started at late stationary phase of the bacterial grown. The maximum lipase activity was obtained in PYB medium after 72 h of growth (Fig 3B). The addition of tributyrin did not increase the lipase activity of the supernatants. For *Terribacillus* sp. 2B122 the maximum lipase activity was also detected in PYB medium at 72 h (Fig 3C). Surprisingly the results of the *Terribacillus* sp. 2B122 lipase activity in LB medium from 48 h to 72 h showed a significantly high error, which may be due to the cell lysis experienced by this strain in LB and LBT at around 24 h of cultivation. Optimal conditions for the lipase activity of *Enterobacter* sp. 1B89 were determined to be maximal in LBT medium at 48 h cultivation. In this case, addition of tributyrin to the culture medium allowed an increase in the lipase activity (Fig 3D).

### Lipase-mediated *O*-deacylation of polyphenols

**Fig 4:**
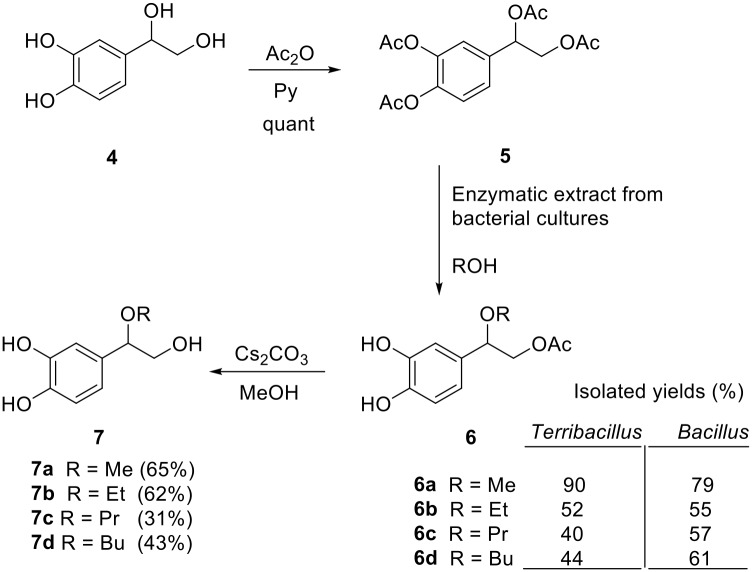
Regioselective deacetylation of peracetylated DHPG (5) with bacterial supernatants.

**Fig 5:**
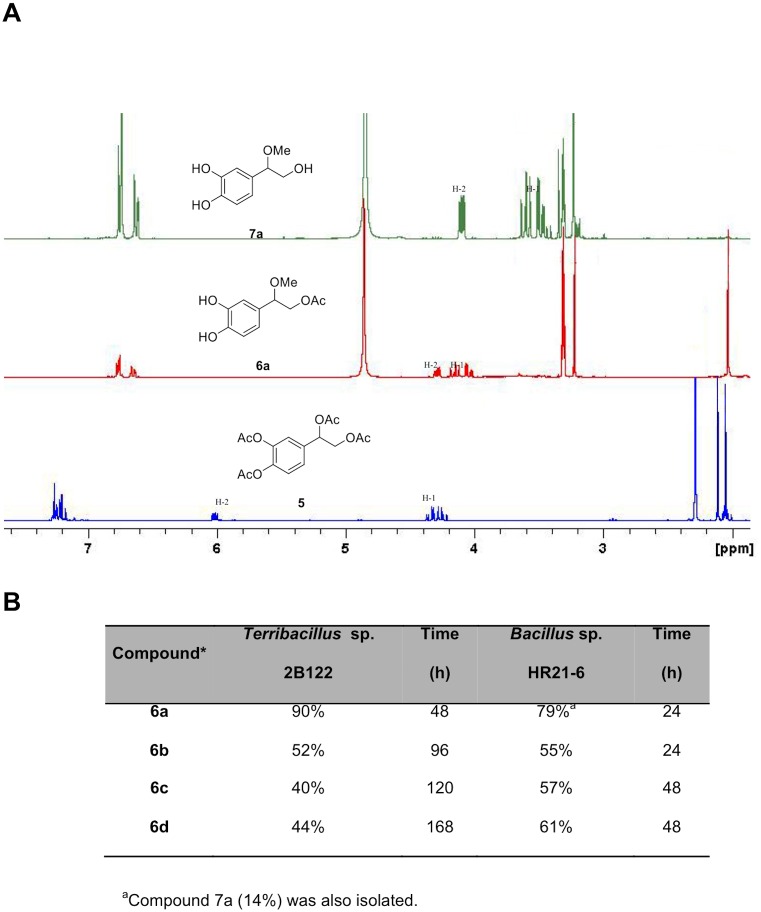
Lipase catalized deacetylation of peracetylated DHPG (5). A) The figure depicts the ^1^H-NMR (300 MHz) spectra of compounds **5** (CDCl_3_), **6a** (CD_3_OD), and **7a** (CD_3_OD). B) Regioselective deacylation of compound **5** using *Terribacillus* sp. 2B122 and *Bacillus* sp. HR21-6 lipases to give compounds 6a-d at the indicated reaction times.

During this work we also aimed at studying another interesting polyphenol, 3,4-dihydroxyphenylglycol (DHPG), which is also present in olives and shows excellent antioxidant characteristics. In a first step, the peracetylated DHPG (compound **5**) was prepared from DHPG **4** [24]. The phenolic hydroxyl groups were regioselectively deacetylated by a transacetylation process with a primary linear aliphatic alcohol (methanol, ethanol, propan-1-ol or butan-1-ol) in a reaction mixture containing the bacterial supernatants and silica gel, using a 1:50:2:2 substrate−alcohol−lipase extract−silica gel ratio. The acetoxy group at the primary position remained unchanged, whereas the acetoxy group at benzylic position was, unexpectedly, substituted by the corresponding alkoxy group (Figs 4 and 5A). The progress of the reactions was monitored by TLC, and it resulted in the synthesis of monoacetylated etherified derivatives **6a-d** (see S1 Supplementary Experimental Procedures) with a yield ranging from 40 to 90% depending on the bacterial isolate (Fig 5B). This procedure constitutes the first synthesis of 3,4-dihydroxyphenylglycol ethers **6**. The enzymes did not only catalyze the alcoholysis of the acetoxy groups on the aromatic ring, but also the substitution of the acetoxy group at benzylic position by an alkoxy group (–OR). The corresponding deacetylated derivatives **7a-d** were prepared by a basic methanolysis in the presence of Cs_2_CO_3_ as a weak base (with a yield of 31–65%, see S1 Supplementary Experimental Procedures). Due to the easy degradation of the catechol group at the high pH required for the deacetylation reaction, sodium ascorbate (1 equiv) was added to the reaction mixture to prevent extensive degradation.

**Fig 6:**
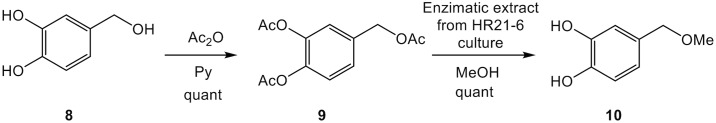
Regioselective deacetylation of peracetylated protocatechuic alcohol (9) catalyzed by the bacterial supernatant of *Bacillus* sp. HR21-6.

The previous reaction on DHPG constituted an outstanding case of an enzymatic conversion of an ester into an ether. In order to further check this reaction, we assayed peracetylated protocatechuic alcohol **9**, which is a compound very similar to peracetylated DHPG **5**, harbouring a benzylic alcohol but lacking the acetoxymethyl group at the end of the aliphatic side chain. Protocatechuic alcohol **8** is a very potent antioxidant molecule, also found in virgin olive oil [25]. As described above, we first obtained the peracetylated derivative of protocatechuic alcohol (compound **9** [26]), and then the deacetylation reaction proceeded in the presence of the bacterial supernatants and MeOH as solvent (Fig 6). Similarly to the reaction described for peracetylated DHPG **5** in Fig 4, the acetoxy group at the benzylic position was substituted by a methoxy group, giving ether **10**.

### Analysis of lipase-mediated *O*-acylation of polyphenols

All the previous reactions were based on the deacetylation of a peracetylated derivative of the corresponding polyphenol. In order to check the selectivity of the enzymes in the bacterial supernatants during esterification reactions, we also tested the direct *O*-acylation of polyphenols **1**, **4** and **8**. In this case, a commercial lipase obtained from *Candida antarctica* (Novozyme 435) used previously in this type of reactions [27] was also studied in parallel with our substrates for comparative purposes. The *O*-acylations of the phenolic compounds **1**, **4** and **8** were carried out with the four lipolytic bacterial extracts and isopropenyl acetate, as solvent and acylating agent, by heating at 40°C for 24 h. The enzymes in the bacterial supernatants were adsorbed on celita for **1** and **8**.

**Fig 7:**
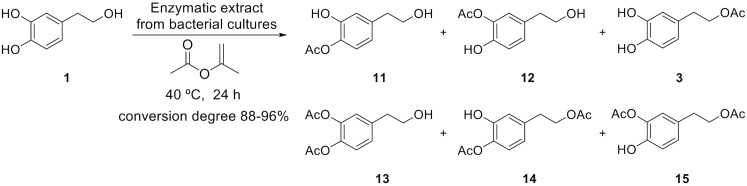
*O*-Acetylation of HT (1) with isopropenyl acetate catalyzed by lipases from the supernatant of bacterial cultures.

The conversion degree for the acetylation of HT (**1**) by the lipase extracts was in the range from 96% for the *Bacillus* sp HR21-6 extract to 88% for the *Enterobacter* sp. 1B89 extract (Fig 7 and Table 1). The OH-4 of the catechol moiety is the more reactive hydroxyl group, and the formation of the monoacetylated derivative at that position (compound **11**) ranged from 44% for *Pseudomonas* sp. 2B120 extract to 34% for *Enterobacter* sp. 1B89 extract. This compound and its regioisomer in position 3 (compound **12**) were formed in a ratio 2:1 after 24 h of reaction (Fig 7 and Table 1). The three diacetylated derivatives **13**−**15** were formed in low proportion (2–10%), and the triacetylated derivative could not be detected (S2 Fig). In contrast, the acetylation was completely regioselective towards the lateral chain with *C*. *antarctica* lipase, yielding only derivative **3** (quant.). Most of the substrate was converted into the acetylated version after 24 h of reaction (88–96% for the bacterial supernatants and 100% for the *Candida* lipase).

**Table 1:** Regioselective acetylation of compounds 1, 4 and 8 with bacterial supernatants or commercial lipase from *C*. *antarctica* (Novozyme 435).

Compound	*Terribacillus* sp. 2B122	*Enterobacter* sp. 1B89	*Pseudomonas* sp. 2B120	*Bacillus* sp. HR21-6	Commercial lipase (*C*. *antarctica*)
**1**	8%	12%	6%	4%	-
**11**	36%	34%	44%	38%	-
**12**	20%	16%	23%	21%	-
**3**	19%	21%	11%	18%	100%
**13**	2%	2%	3%	3%	-
**14**	10%	9%	5%	9%	-
**15**	5%	6%	8%	7%	-
**4**	39%	30%	32%	34%	47%
**16**	17%	16%	14%	12%	10%
**17**	16%	16%	13%	11%	10%
**18**	15%	18%	22%	23%	20%
**19**	7%	11%	10%	11%	7%
**20**	7%	9%	9%	9%	6%
**8**	22%	12%	10%	10%	5%
**21**	22%	24%	35%	27%	-
**22**	19%	20%	29%	23%	-
**23**	20%	24%	13%	21%	87%
**24**	3%	1%	2%	1%	-
**25**	7%	10%	6%	9%	9%
**26**	7%	9%	5%	9%	-

**Fig 8:**
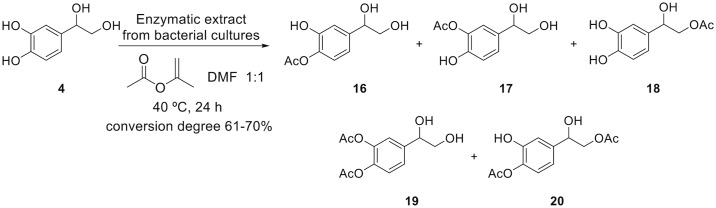
*O*-Acetylation of DHPG (4) with isopropenyl acetate catalyzed by lipases from the supernatant of bacterial cultures.

When the acetylation reaction was carried out on DHGP (compound **4**), both monoacetylated and diacetylated derivatives were also obtained (Fig 8 and S2D and S2E Fig). A low conversion degree for the acetylation of **4** is found for the lipase extracts, in a range of from 61% to 70% (Table 1). The monoacetylated derivatives in the catechol moiety **16**, **17** and in the primary aliphatic alcohol **18** were formed in similar proportions (23–11%). The ratios between the monoacylation in the aromatic ring and the aliphatic chain were 1:1:1 when the supernatants from *Terribacillus* sp. 2B122 or *Enterobacter* sp. 1B89 were used, and 1:1:2 when the supernatants from *Pseudomonas* sp. 2B120 or *Bacillus* sp. HR21-6 were employed (Table 1). Surprisingly, no acylation of the benzylic hydroxyl group was observed in any case (Fig 8 and S2D and S2E Fig). The acylation in the presence of the *C*. *antarctica* lipase showed a low regioselectivity, and the monoacylation in the primary hydroxyl group only took place with a 20% yield. Remarkably the conversion of the original substrate achieved with the lipase from *C*. *antarctica* was lower (53%) than those with the bacterial lipases extracts (61–70%).

**Fig 9:**
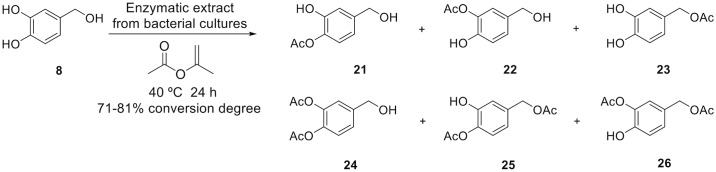
*O*-Acetylation of protocatechuic alcohol (8) with isopropenyl acetate catalyzed by lipases from the supernatant of bacterial cultures.

Since it was very interesting that the benzylic alcohol in DHPG was not acetylated, we also tested the protocatechuic alcohol **8** for regioselective acetylation of the benzylic alcohol. In this case, a mixture of all the possible mono- and di-acetylated derivatives, including the benzylic position, was observed regardless of the bacterial isolate (Fig 9 and S2F–S2H Fig). The OH-4 of the catechol moiety was the most reactive hydroxyl group of **8** (Fig 9), and the monoacetylation at that position ranged from 35% to 22%. The ratio of the three possible monoacetylated compounds **21–23** was different depending on the isolate: 1:1:1 in the case of *Terribacillus* sp. 2B122, 1:2:2 in the case of *Enterobacter* sp. 1B89, 1:3.5:3 in the case of *Pseudomonas* sp. 2B120, and approx. 1:3:2 in the case of *Bacillus* sp. HR21-6. The degree of conversion of the protochatechuic alcohol **8** (78–90%) was lower than the HT **1** (88–96%) but higher than the DHGP **4** (61–70%). The three diacetylated derivatives **24–26** were formed in low proportion (1–10%) and triacetylated derivative **9** was not detected as happened with HT and DHPG. The acylation with *C*. *antarctica* lipase is highly regioselective towards the lateral chain to form **23**, although less efficient than the acylation of **1**.

### DPPH radical scavenging activity assay

**Fig 10:**
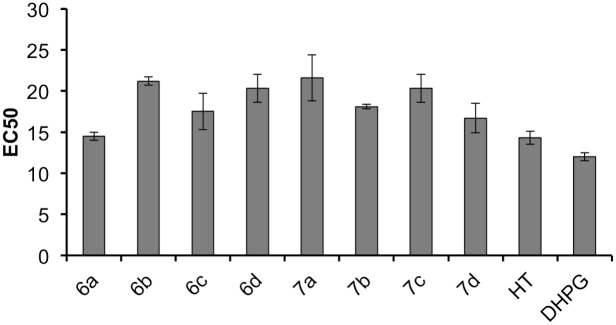
DPPH radical scavenging activity of lipophilic derivatives of DHPG (compounds 6a-d and 7a-d) compared to HT and DHPG.

DHPG displays a strong antioxidant activity. Acetylation of the non-catecholic alcohols increases its lypophilic character, which expands the possibilities of formulations for this compound in the cosmetics, food and pharmacology industries. Therefore, the antioxidant capabilities of phenolic compounds **6a-d** and **7a-d** was evaluated using the DPPH method [19,20]. The EC_50_ value was calculated through the lineal representation of each compound in 5 different concentrations between 5 and 25 μM, and it represents the concentration of the compound necessary to reduce the amount of the DPPH radical to 50%, i.e. lower concentrations of the compound under test indicate a higher antioxidant capacity. For comparative purposes, HT **1** and DHPG **4**, two antioxidants with remarkable activity were included. As shown in Fig 10, all the compounds have a similar antiradical activity (14–21 μM). In addition, the compounds **6a** and **7d** showed the best activity with values comparable to that of HT (14.5 ± 0.5 and 16.7 ± 1.8 μM, respectively).

## Discussion

Previous reports have shown that the lipophilic derivatives and analogs of one of the olive phenols, HT, with esters or ethers functionalities display a better hydrophilic/lipophilic balance [28], an increased bioavailability compared with their unprotected analogs [29] and stability to oxygen compared with the underivatized HT, which make these molecules more attractive for the food industry [30]. These findings prompted us to develop three innovative aspects. Firstly, we introduce a novel concept of biocatalyst for the widespread use in industry, employing extracts of lipolytic bacterial strains. These extracts were only partially purified and consequently very cheap to obtain, but displaying performance levels similar or even higher than commercially available purified lipases. Additionally, the use of these lipolytic extracts allowed us to obtain unprecedented analogs in one single step.

Secondly, in this study, in addition to HT, we have also employed DHPG, a compound also present in olives [23]. Indeed, our data confirmed that DHPG is more efficient as antioxidant than HT. DHPG is a simple phenol structurally similar to HT, but with an additional hydroxyl group at the benzylic position. The reason to use this poorly studied polyphenol relies on its antioxidant efficiency in water, which is 2–3 times higher than that of ascorbic acid or HT, whereas in lipid medium it is comparable to that of vitamin E despite its high polarity [31]. Interestingly, this compound could be obtained from olive-mill wastes (alperujo) using a simple method [32]. The wastes of the olive oil industry are considered a cheap and easily available source of bioactive compounds, whose activity is reflected in the olive oil itself. DHPG displays excellent possibilities to be modified, containing different free OH groups on its structure that can be acylated, obtaining a battery of novel bioactive lipophilic derivatives with improved activity and bioavailability. Despite of its advantages, to the best of our knowledge this compound has never been reported as an ingredient for the formulation of functional foods, and no semisynthetic derivatives of this compound have been reported in the literature. During this work, the deacetylation of fully acetylated DHPG **5** in different aliphatic alcohols gave not only deprotection of the phenolic hydroxyl groups, but also an unexpected substitution of the acetoxy group at the benzylic position by an alkoxy group, which allowed access in one single synthetic step to a new family of lipophilic polyphenols **6a-6d**, bearing both ester and ether functionalities, that can be tailored to get the best pharmacological profile.

**Fig 11:**
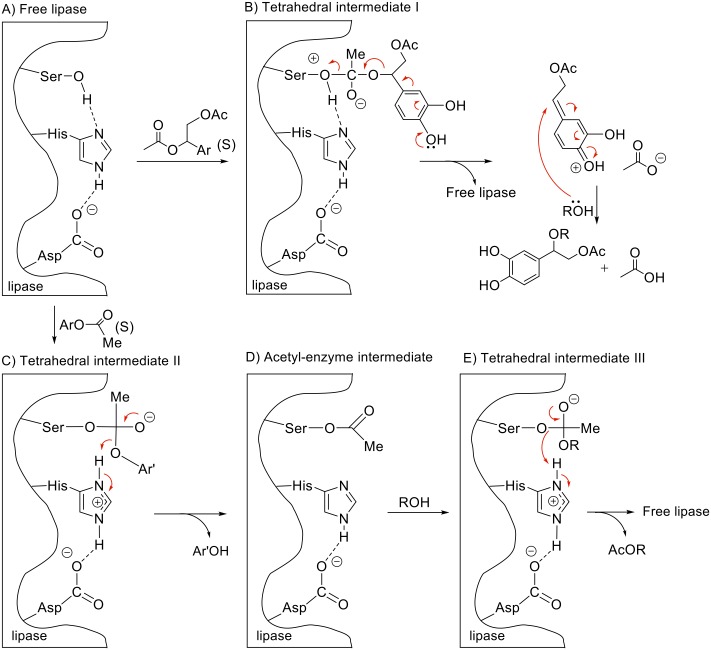
Proposed mechanism for the lipase catalysed formation of 3,4-dihydroxyphenylglycol ethers 6.

A generally accepted mechanism for lipase-catalyzed transesterification reactions involves Ser, His and Asp residues as the catalytic triad, and the intermediates II and III shown in Fig 11 [33]. For the alcoholysis of the acetate group at the benzylic position, the tetrahedral intermediate I might be the key step. The electron donating character of the *p*-hydroxyl group helps to eliminate the acetate group simultaneously to the release of the free enzyme. Finally, the stabilized oxocarbenium cation reacts at the benzylic position with ROH, what might explain the outcome of the reaction.

An increase in the reaction rate and the complete disappearance of the starting material was observed when silica gel was added to the reaction mixture. On the contrary, using only MeOH and lipase we observed a slower reaction rate and a low final conversion. These results are in agreement with the significant improvement of the conversion yield when silica gel-adsorbed substrates were used in lipase-catalyzed esterification reactions [34]. The critical role of silica gel in these kinds of reactions was associated with its behavior as polar substrate reservoir and with the protection of the enzyme avoiding its blockage [35]

Furthermore, ethers **7a-7d** bearing a free hydroxyl group at the end of the side chain were easily accessed by chemical hydrolysis of the acetate of **6a-6d**, expanding even more the possibilities for these compounds. Some of these compounds have already been detected before. In particular, compound **7a** was detected in olive (*Olea europaea*) tree leaves [36] and compound **10** in the fermentation broth of the marine fungus Y26-02 [18].

And lastly, and also to the best of our knowledge it is the first time that a lipase mediated reaction has catalyzed not only the deacylation of phenolic esters, but also the deacetoxylation of an ester group at benzylic position. At the same time, the aliphatic ester at the end of the side chain of peracetylated DHPG **5** remained untouched. On the contrary, peracetylated hydroxytyrosol **2** could not be transformed into ether because it lacks the acetoxy group at the right position. However, a series of HT alkyl ethers, with an alkoxy group at the end of the side chain, have been previously prepared in three steps starting from HT [37]. These methyl, ethyl, propyl and butyl ethers reduced ROS generation, malondialdehyde formation, and glutathione depletion in HepG2 human hepatocarcinoma cells treated [38]. Similarly, HT alkyl ethers (C2-C12) inhibited lipid peroxidation and reduced glutathione depletion in rat brain slices, after hypoxia and re-oxygenation [39]. Esters and ethers derived from HT exert higher anti-proliferative and pro-apoptotic activity than HT itself. The highest cytotoxic activity was found by the ethers with the longest alkyl chain [40]. Similarly, these lipophilic derivatives inhibit both monocyte and macrophage pro-tumorigenic inflammatory activities and platelet function in a more effective way than HT [41], which reflects the broad applicability and importance of these type of molecules.

In conclusion, this study opens new avenues for the food industries to obtain unprecedented derivatives of antioxidants with expanded physico-chemical properties and utilities. Families of DHPG derivatives could be more suited for these purposes than the extensively studied HT. Consequently, biological and pharmacological studies of both series of DHPG derivatives will be undertaken, in order to elucidate the more effective compounds in cancer and chronic degenerative disease prevention, and as anti-inflammatory compounds.

## Supporting Information

S1 Fig Phylogenetic maximum-likelihood tree showing the position of *Bacillus* sp HR21-6 with respect to other type strains of the genus *Bacillus*. The 16S rRNA sequence of *Streptococcus pneumoniae* was used as an external group. 16S rRNA gene sequences from the isolates correspond to 1380 bps. The phylogenetic reconstruction carried out with different methods was consistent, and consequently only the tree obtained with Neighbor-Joining, for the evolutionary history, and Maximum Composite Likelihood, for evolutionary distances is shown.
(TIF)

S2 Fig Lipase catalized acetylation of polyphenols HT (1), DHPG (4) and protocatechuic alcohol (8). Partial ^1^H-NMR spectra for the lipase-mediated acetylation of HT (A) to give mono- and di-acetylated derivatives of HT (C), compared to the peracetylated HT (B). Partial ^1^H-NMR spectra for the lipase-mediated acetylation of DHPG (D) to give mono- and di-acetylated derivatives of DHPG (E). Partial ^1^H-NMR spectra for the lipase-mediated acetylation of protocatechuic alcohol (F) to give mono- and di-acetylated derivatives of protocatechuic alcohol (H), compared to the peracetylated protocatechuic alcohol (G).
(TIF)

S1 Supplementary Experimental Procedures 2-(3,4-Dihydroxyphenyl)-2-metoxyethyl acetate (**6a**) from compound **5;** 2-(3,4-Dihydroxyphenyl)-2-ethoxyethyl acetate (**6b**); 2-(3,4-Dihydroxyphenyl)-2-propoxyethyl acetate (**6c**); 2-Butoxy-2-(3,4-dihydroxyphenyl)ethyl acetate (**6d**); 2-(3,4-Dihydroxyphenyl)-2-methoxyethanol (**7a**); 2-(3,4-Dihydroxyphenyl)-2-ethoxyethanol (**7b**); 2-(3,4-Dihydroxyphenyl)-2-propoxyethanol (**7c**); 2-Butoxy-2-(3,4-dihydroxyphenyl)ethanol (**7d**); Spectroscopic data for crude reaction depicted in Fig 7; Spectroscopic data for crude reaction depicted in Fig 8; Spectroscopic data for crude reaction depicted in Fig 9.
(PDF)

S1 Table Selection of lipolytic microorganisms in the primary screening (hydrolytic). (PDF)

S2 Table Absorbance values obtained in the transesterification assay. In red, the isolates selected with absorbance values higher than the highest hydrolysis control.
(PDF)
