# Correction: A Straightforward Access to New Families of Lipophilic Polyphenols by Using Lipolytic Bacteria

**DOI:** 10.1371/journal.pone.0307130

**Published:** 2024-07-10

**Authors:** Leyre Sánchez-Barrionuevo, Alejandro González-Benjumea, Almudena Escobar-Niño, María Teresa García, Óscar López, Inés Maya, José G. Fernández-Bolaños, David Cánovas, Encarnación Mellado

In the Funding section, the grant number associated with the funding received from the Spanish Ministerio de Economía y Competitividad is missing. The grant number is: BIO2015-67148-R.
