# Supplementary figures and images for: A Straightforward Access to New Families of Lipophilic Polyphenols by Using Lipolytic Bacteria
Source: PLoS One. 2016 Nov 17;11(11):e0166561. doi: 10.1371/journal.pone.0166561 (PMC5113952; doi:10.1371/journal.pone.0166561)

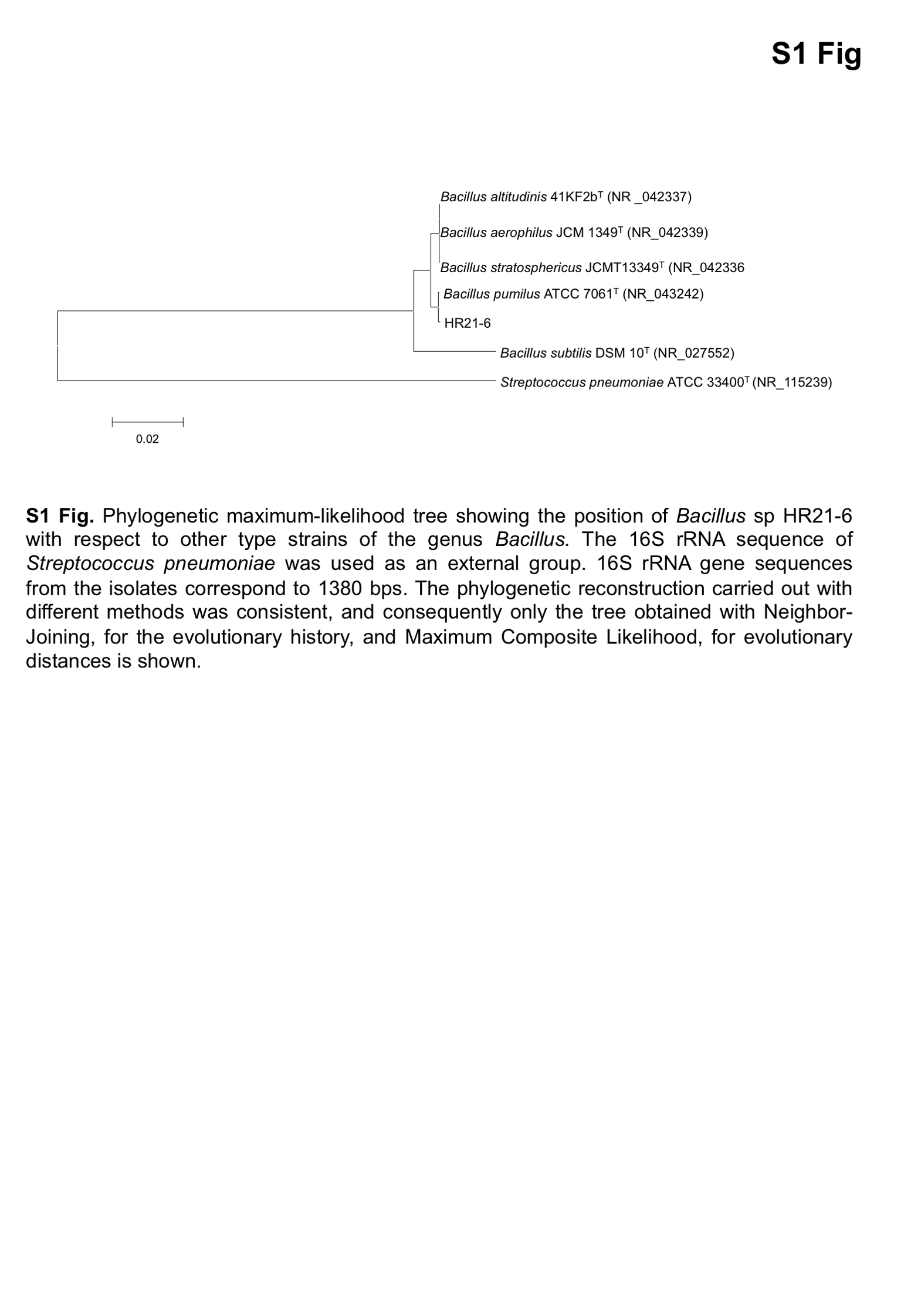

Supplement: S1 Fig — The 16S rRNA sequence of Streptococcus pneumoniae was used as an external group. 16S rRNA gene sequences from the isolates correspond to 1380 bps. The phylogenetic reconstruction carried out with different methods was consistent, and consequently only the tree obtained with Neighbor-Joining, for the evolutionary history, and Maximum Composite Likelihood, for evolutionary distances is shown. (TIF) [file pone.0166561.s001.tif]

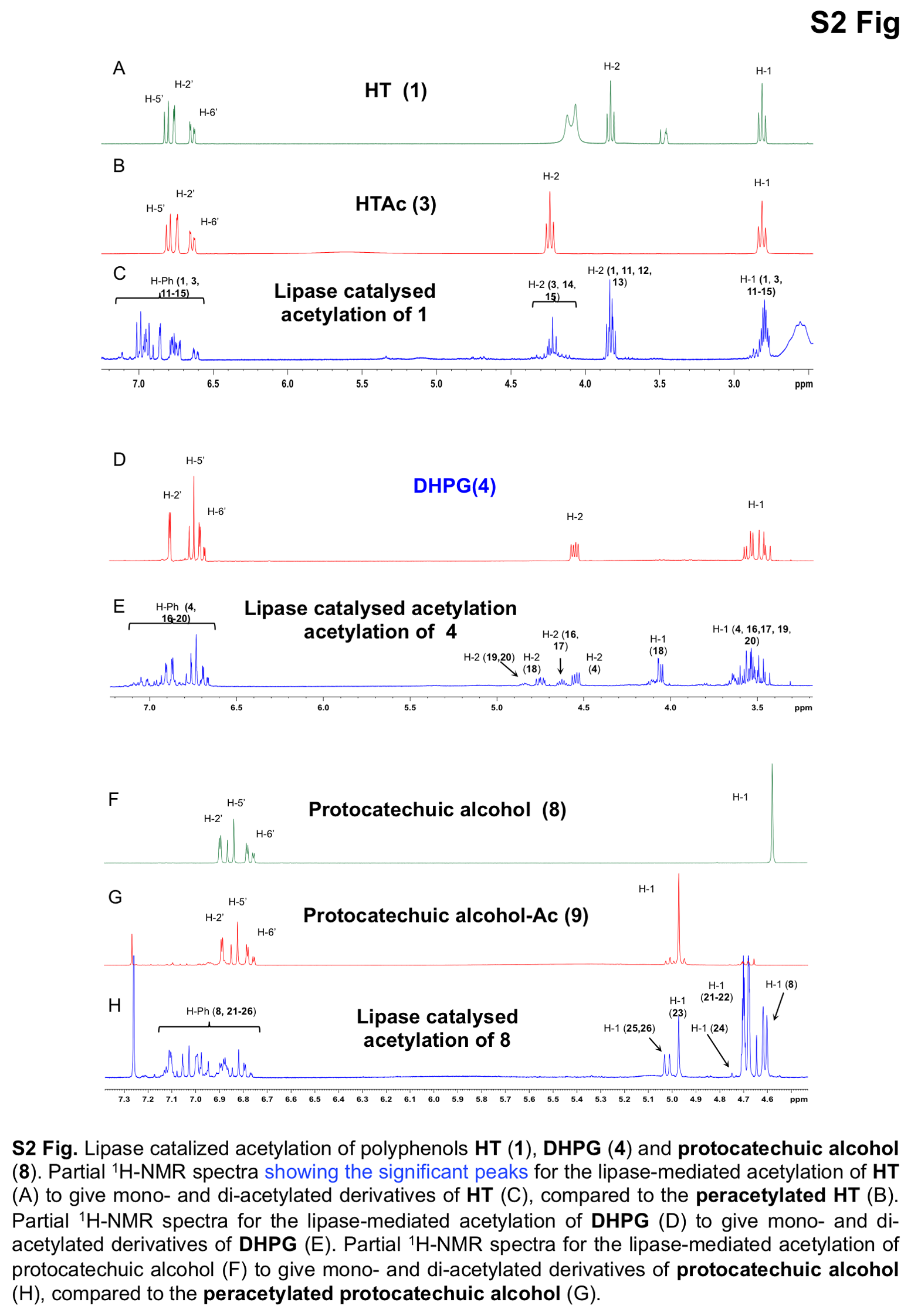

Supplement: S2 Fig — Partial 1H-NMR spectra for the lipase-mediated acetylation of HT (A) to give mono- and di-acetylated derivatives of HT (C), compared to the peracetylated HT (B). Partial 1H-NMR spectra for the lipase-mediated acetylation of DHPG (D) to give mono- and di-acetylated derivatives of DHPG (E). Partial 1H-NMR spectra for the lipase-mediated acetylation of protocatechuic alcohol (F) to give mono- and di-acetylated derivatives of protocatechuic alcohol (H), compared to the peracetylated protocatechuic alcohol (G). (TIF) [file pone.0166561.s002.tif]
