# Supplementary material for: A Straightforward Access to New Families of Lipophilic Polyphenols by Using Lipolytic Bacteria
Source: PLoS One. 2016 Nov 17;11(11):e0166561. doi: 10.1371/journal.pone.0166561 (PMC5113952; doi:10.1371/journal.pone.0166561)
Supplement: S1 Supplementary Experimental Procedures — 2-(3,4-Dihydroxyphenyl)-2-metoxyethyl acetate (6a) from compound 5; 2-(3,4-Dihydroxyphenyl)-2-ethoxyethyl acetate (6b); 2-(3,4-Dihydroxyphenyl)-2-propoxyethyl acetate (6c); 2-Butoxy-2-(3,4-dihydroxyphenyl)ethyl acetate (6d); 2-(3,4-Dihydroxyphenyl)-2-methoxyethanol (7a); 2-(3,4-Dihydroxyphenyl)-2-ethoxyethanol (7b); 2-(3,4-Dihydroxyphenyl)-2-propoxyethanol (7c); 2-Butoxy-2-(3,4-dihydroxyphenyl)ethanol (7d); Spectroscopic data for crude reaction depicted in Fig 7; Spectroscopic data for crude reaction depicted in Fig 8; Spectroscopic data for crude reaction depicted in Fig 9. (PDF) [file pone.0166561.s003.pdf]

S1 Supplementary Experimental Procedures.

2-(3,4-Dihydroxyphenyl)-2-metoxyethyl acetate (6a) from compound 5

Eluted with CH_2Cl_2-MeOH 40:1 → 10:1 gave **6a**, 90% with *Terribacillus* sp. 2B122 and 79% with *Bacillus* sp. HR21-6. ^{1}H-NMR (300 MHz, CD_3OD): δ 6.80 (d 1H, $J_{5,6}$ = 8.0 Hz, Ar-5), 6.79 (d 1H, $J_{2,6}$ = 2.0 Hz, Ar-2), 6.68 (dd 1H, $J_{6,5}$ = 8.0 Hz, $J_{6,2}$ = 2.0 Hz, Ar-6), 4.32 (dd 1H, $J_{2,1a}$ = 8.0 Hz, $J_{2,1b}$ = 3.9 Hz, CH), 4.18 and 4.07 (dd 2H each, $J_{1a,1b}$ = 11,4 Hz, $J_{1a,2}$ = 8.0 Hz, $J_{1b,2}$ = 3.9 Hz, CH_2), 3.25 (s 3H, OMe), 2.06 (s 3H, Ac); ^{13}C-NMR (75.5 MHz, CD_3OD): δ 173.6 (CO), (Ar-3 and 4), 131.4 (Ar-1), 120.7 (Ar-6), 117.2 (Ar-5), 115.8 (Ar-2), 83.4 (CH), 69.5 (CH_2), 57.7 (OMe), 21.6 (Ac); IR (cm^{-1}): 1389, 1367, 1235, 1114, 1036. HRCI-MS *m/z*: calcd for $C_{11}H_{14}O_5$ ($[M]^+$): 226.0841, found 226.0845.

2-(3,4-Dihydroxyphenyl)-2-ethoxyethyl acetate (6b)

Eluted with CH_2Cl_2-EtOH 40:1 gave **6b**, 52% with *Terribacillus* sp. 2B122 and 55% with *Bacillus* sp. HR21-6. ^{1}H-NMR (300 MHz, $CDCl_3$): δ 6.89 (d 1H, $J_{2,6}$ = 2.0 Hz, Ar-2), 6.83 (d 1H, $J_{5,6}$ = 8.1 Hz, Ar-5), 6.72 (dd 1H, $J_{6,5}$ = 8.1 Hz, $J_{6,2}$ = 2.0 Hz, Ar-6), 4.42 (dd 1H, $J_{2,1a}$ = 6.9 Hz, $J_{2,1b}$ = 5.0 Hz, CH), 4.20-4.12 (m 2H, CH_2), 3.43 (cc 2H, $J_{1'a,1'b}$ = 16.4 Hz, $J_{1',2'}$ = 9.4 Hz, CH_2 (OEt)), 2.06 (s 3H, Ac), 1.17 (t 3H, $J_{2',1'}$ = 7.0 Hz, CH_3 (OEt)); ^{13}C-NMR (75.5 MHz, $CDCl_3$): δ 172.0 (CO), 144.5 (Ar-4), 144.4 (Ar-3), 131.0 (Ar-1), 119.9 (Ar-6), 115.5 (Ar-5), 113.9 (Ar-2), 79.6 (CH), 68.4 (CH_2), 64.8 (CH_2 (OEt)), 21.3 (Ac), 15.4 (CH_3 (OEt)); IR (cm^{-1}): 3368, 1713, 1232, 1109, 1035; HRCI-MS *m/z*: calcd for $C_{12}H_{16}O_5$ ($[M]^+$): 240.0998, found 240.0990.

2-(3,4-Dihydroxyphenyl)-2-propoxyethyl acetate (6c)

Eluted with EtOAc-hexane 1:3 → 1:1 gave **6c** 40% with *Terribacillus* sp. 2B122 and 57% with *Bacillus* sp. HR21-6. ^{1}H-NMR (300 MHz, $CDCl_3$): δ 6.90 (d 1H, $J_{2,6}$ = 2.0 Hz, Ar-2), 6.84 (d 1H, $J_{5,6}$ = 8.1 Hz, Ar-5), 6.73 (dd 1H, $J_{6,5}$ = 8.1 Hz, $J_{6,2}$ = 2.0 Hz, Ar-6), 4.41 (dd 1H, $J_{2,1a}$ = 7.6 Hz, $J_{2,1b}$ = 4.5 Hz, CH), 4.21-4.10 (m 2H, CH_2), 3.40-3.24 (m 2H, OCH_2 (OPr)), 2.06 (s 3H, Ac), 1.59 (sext 2H, J = 7.4 Hz, CH_2 (OPr)), 0.88 (t 3H, J = 7.4 Hz, CH_3 (OPr)); ^{13}C-NMR (75.5 MHz, $CDCl_3$): δ 171.9 (CO), 144.4 (Ar-4), 144.3 (Ar-3), 131.2 (Ar-1), 120.0 (Ar-6), 115.5 (Ar-5), 113.9 (Ar-2), 79.7 (CH), 71.1 (CH_2), 68.4 (OCH_2 (OPr)), 23.1 (CH_2 (OPr)), 21.3 (Ac), 10.8 (CH_3 (OPr)); IR (cm^{-1}): 3368, 1714, 1230, 1109, 1003; HRCI-MS *m/z*: calcd for $C_{13}H_{18}O_5$ ($[M]^+$): 254.1154, found 254.1148.

2-Butoxy-2-(3,4-dihydroxyphenyl)ethyl acetate (6d)

Eluted with EtOAc-hexane 1:2 gave **6d**, 44% with *Terribacillus* sp. 2B122 and 61% with *Bacillus* sp. HR21-6. ^{1}H-NMR (300 MHz, $CDCl_3$): δ 6.90 (d 1H, $J_{2,6}$ = 1.9 Hz, Ar-2), 6.84 (d 1H, $J_{5,6}$ = 8.0 Hz, Ar-5), 6.73 (dd 1H, $J_{6,5}$ = 8.0 Hz, $J_{6,2}$ = 1.9 Hz, Ar-6), 6.50 (s 1H, OH), 5.95 (s 1H, OH), 4.44 (dd 1H, $J_{2,1a}$ = 7.7 Hz, $J_{2,1b}$ = 4.4 Hz, CH), 4.22-4.10 (m 2H, CH_2), 3.45-3.28 (m 2H, $OC\mathit{H_2}CH_2CH_2CH_3$), 2.07 (s 3H, Ac), 1.55 (c 2H, J = 6.6 Hz, $OCH_2C\mathit{H_2}CH_2CH_3$), 1.39-1.25 (m 2H, $OCH_2CH_2C\mathit{H_2}CH_3$), 0.86 (t 3H, J = 7.3 Hz, $OCH_2CH_2CH_2C\mathit{H_3}$); ^{13}C-NMR (75.5 MHz, $CDCl_3$): δ 172.1 (CO), 144.4 (Ar-4), 144.3 (Ar-3), 131.1 (Ar-1), 120.0 (Ar-6), 115.5 (Ar-5), 113.9 (Ar-2), 79.7 (CH), 69.2 ($O\mathit{C}H_2CH_2CH_2CH_3$), 68.4 ($CH_2$), 32.0 ($OCH_2\mathit{C}H_2CH_2CH_3$), 21.3 (Ac), 19.6 ($OCH_2CH_2\mathit{C}H_2CH_3$), 14.2 ($OCH_2CH_2CH_2\mathit{C}H_3$); IR ($cm^{-1}$): 3361, 2960, 1711, 1232, 1036; HRCI-MS *m/z*: calcd for $C_{14}H_{20}O_6$ ($[M]^+$): 268.1311, found 268.1320.

2-(3,4-Dihydroxyphenyl)-2-methoxyethanol (7a)

Eluted with CH_2Cl_2-MeOH 1:0 → 1:20 gave **7a**, 65%. ^{1}H-NMR (300 MHz, D_2O): δ 6.94 (d 1H, $J_{5,6}$ = 8.1 Hz, Ar-5), 6.89 (d 1H, $J_{2,6}$ = 2.0 Hz, Ar-2), 6.80 (dd 1H, $J_{6,5}$ = 8.1 Hz, $J_{6,2}$ = 2.0 Hz, Ar-6), 4.80 (s 3H, OH), 4.29 (dd 1H, $J_{1',2'a}$ = 7.5 Hz, $J_{1',2'b}$ = 4.6 Hz, CH), 3.71 and 3.64 (2 dd 1H each, $J_{2'a,2'b}$ = 11.9 Hz, $J_{2'a,1'}$ = 7.5 Hz, $J_{2'b,1'}$ = 4.6 Hz, CH_2), 3.27 (s 3H, OMe); ^{13}C-NMR (75.5 MHz, D_2O): δ 144.1 (Ar-1), 144.0 (Ar-2), 130.5 (Ar-4), 119.8 (Ar-5), 116.1 (Ar-6), 114.7 (Ar-3), 83.6 (CH), 65.0 (CH_2), 56.0 (OMe); IR (cm^{-1}): 3315, 1520, 1281, 1198, 1104, 1064, 1029; HRCI-MS *m/z*: calcd for $C_9H_{12}O_4$ ([M]$^+$): 184.0736, found 184.0735.

2-(3,4-Dihydroxyphenyl)-2-ethoxyethanol (7b)

Eluted with CH_2Cl_2-EtOH 1:0 → 1:10 gave **7b**, 62%. ^{1}H-NMR (300 MHz, CD_3OD): δ 6.91 (d 1H, $J_{5,6}$ = 8.1 Hz, Ar-5), 6.88 (d 1H, $J_{2,6}$ = 1.8 Hz, Ar-2), 6.80 (dd 1H, $J_{6,5}$ = 8.1 Hz, $J_{6,2}$ = 2.0 Hz, Ar-6), 4.84 (s 3H, OH), 4.38 (dd 1H, $J_{1',2'a}$ = 7.7 Hz, $J_{1',2'b}$ = 4.4 Hz, CH), 3.70 and 3.61 (2 dd 1H each, $J_{2'a,2'b}$ = 11.7 Hz, $J_{2'a,1'}$ = 8.0 Hz, $J_{2'b,1'}$ = 4.4 Hz, CH_2), 3.48 (c 2H, J = 6.9 Hz, CH_2 (OEt)), 1.19 (t 3H, J = 7.1 Hz, CH_3 (OEt)); ^{13}C-NMR (75.5 MHz, CD_3OD): δ 146.1 (Ar-4), 145.9 (Ar-3), 133.1 (Ar-1), 121.3 (Ar-6), 117.7 (Ar-5), 116.3 (Ar-2), 83.9 (CH), 67.3 (CH_2), 66.3 (CH_2 (OEt)), 16.1 (CH_3 (OEt)); IR (cm^{-1}): 3347, 1280, 1092, 1082; HRCI-MS *m/z*: calcd for $C_{10}H_{14}O_4$ ([M]$^+$): 199.0970, found 199.0972.

2-(3,4-Dihydroxyphenyl)-2-propoxyethanol (7c)

Eluted with EtOAc-hexane 1:1 gave **7c**, 31%. ^{1}H-NMR (300 MHz, CD_3OD): δ 6.78 (d 1H, $J_{2,6}$ = 1.9 Hz, Ar-2), 6.76 (d 1H, $J_{5,6}$ = 8.0 Hz, Ar-5), 6.65 (dd 1H, $J_{6,5}$ = 8.0 Hz, $J_{6,2}$ = 2.0 Hz, Ar-6), 4.86 (s 3H, OH), 4.22 (dd 1H, $J_{1',2'a}$ = 8.0 Hz, $J_{1',2'b}$ = 4.1 Hz, CH), 3.64

and 3.51 (2 dd 1H each, $J_{2'a,2'b}$ = 11.6 Hz, $J_{2'a,1'}$ = 8.0 Hz, $J_{2'b,1'}$ = 4.1 Hz, CH_2), 3.37-3.29 (m 2H, CH_2 (OPr)), 1.61 (s 2H, J = 7.2 Hz, CH_2 (OPr)), 0.93 (t 3H, J = 7.4 Hz, CH_3 (OPr)); ^{13}C-NMR (75.5 MHz, CD_3OD): δ 147.3 (Ar-4), 146.9 (Ar-3), 133.3 (Ar-1), 120.6 (Ar-6), 117.0 (Ar-5), 115.8 (Ar-2), 85.2 (CH), 72.5 (CH_2), 68.9 (OCH_2 (OPr)), 24.9 (CH_2 (OPr)), 11.8 (CH_3 (OPr)); IR (cm^{-1}): 3338, 2928, 1281, 1101, 1021; HRCI-MS *m/z*: calcd for $C_{11}H_{16}O_4Na$ ($[M + Na]^+$): 235.0941, found 235.0939.

2-Butoxy-2-(3,4-dihydroxyphenyl)ethanol (7d)

Eluted with EtOAc-hexane 1:1 gave **7d**, 43%. ^{1}H-NMR (300 MHz, $CDCl_3$): δ 6.84 (d 1H, $J_{2,6}$ = 1.8 Hz, Ar-2), 6.83 (d 1H, $J_{5,6}$ = 8.0 Hz, Ar-5), 6.72 (dd 1H, $J_{6,5}$ = 8.0 Hz, $J_{6,2}$ = 1.8 Hz, Ar-6), 6.04 (s 3H, OH), 4.29 (dd 1H, $J_{2,1a}$ = 7.8 Hz, $J_{2,1b}$ = 4.4 Hz, CH), 3.69-3.57 (m 2H, CH_2), 3.47-3.28 (m 2H, $OCH_2CH_2CH_2CH_3$), 1.60-1.51 (m 2H, $OCH_2CH_2CH_2CH_3$), 1.43-1.26 (m 2H, $OCH_2CH_2CH_2CH_3$), 0.88 (t 3H, J = 7.3 Hz, $OCH_2CH_2CH_2CH_3$); ^{13}C-NMR (75.5 MHz, $CDCl_3$): δ 144.3 (Ar-4), 144.2 (Ar-3), 131.8 (Ar-1), 119.9 (Ar-6), 115.7 (Ar-5), 114.1 (Ar-2), 82.5 (CH), 69.2 ($OCH_2CH_2CH_2CH_3$), 67.6 (CH_2), 32.2 ($OCH_2CH_2CH_2CH_3$), 19.7 ($OCH_2CH_2CH_2CH_3$), 14.2 ($OCH_2CH_2CH_2CH_3$); IR (cm^{-1}): 3317, 2956, 1281, 1098, 1023; HRCI-MS *m/z*: calcd for $C_{12}H_{18}O_4Na$ ($[M + Na]^+$): 249.1097, found 249.1087.

Spectroscopic data for crude reaction depicted in Scheme 4

^{1}H-NMR (700 MHz, $CDCl_3$): δ 7.11 (m, 2H, H-5', H-6' **13**), 7.06 (m, 1H, H-2' **13**), 7.02 (d, 1H, J= 8.3 Hz, H-5' **14**), 7.01 (d, 1H, J= 8.2 Hz, H-5' **11**), 6.99 (dd, 1H, J= 8.2 Hz, J= 2.1 Hz, H-6' **12**), 6.98 (dd, J= 8.2 Hz, J= 2.1 Hz, H-6' **15**), 6.96 (d, 1H, J= 2.1 Hz, H-2' **12**), 6.95 (d, 1H, J= 2.1 Hz, H-2' **15**), 6.93 (d, 1H, J= 8.3 Hz, H-5' **12**), 6.92 (d, 1H, J= 8.3 Hz, H-5' **15**), 6.87 (d, 1H, J= 2.1 Hz, H-2' **11**), 6.86 (d, 1H, J= 2.1 Hz, H-2' **14**), 6.80 (d, 1H, J= 8.3 Hz, H-5' **3**), 6.79 (dd, J= 8.2 Hz, J= 2.1 Hz, H-6' **14**), 6.78 (d, 1H, J= 8.3 Hz, H-5' **1**), 6.77 (dd, J= 8.2 Hz, J= 2.1 Hz, H-6' **11**), 6.74 (d, 1H, J= 2.1

Hz, H-2’ **1**), 6.73 (d, 1H, *J*= 2.1 Hz, H-2’ **3**), 6.64 (dd, *J*= 8.2 Hz, *J*= 2.1 Hz, H-6’ **1**), 6.62 (dd, *J*= 8.2 Hz, *J*= 2.1 Hz, H-6’ **3**), 4.26, 4.24 (2t, 2H each, *J*= 7.1 Hz, H-1 **14**, **15**), 4.22 (t, 2H, *J*= 7.1 Hz, H-1 **3**), 3.86 (t, 2H, *J*= 7.1 Hz, H-1 **13**), 3.84 (t, 2H, *J*= 7.1 Hz, H-1 **11**), 3.82 (t, 2H, *J*= 7.1 Hz, H-1 **12**), 3.81 (t, 2H, *J*= 7.1 Hz, H-1 **1**), 2.92 (t, 2H, *J*= 7.1 Hz, H-2 **13**), 2.87 (t, 2H, *J*= 7.1 Hz, H-2 **14**), 2.85 (t, 2H, *J*= 7.1 Hz, H-2 **15**), 2.81 (t, 2H, *J*= 7.1 Hz, H-2 **3**), 2.80 (t, 2H, *J*= 7.1 Hz, H-2 **11**), 2.79 (t, 2H, *J*= 7.1 Hz, H-2 **12**), 2.75 (t, 2H, *J*= 7.1 Hz, H-2 **1**), 2.35, 2.34, 2.28 (4s, 3H each, ArOCO*Me* **11-15**), 2.04, 2.03 (3s, 3H each, OCO*Me* **3**, **14**, **15**).

^{13}C-NMR (175 MHz, $CDCl_3$): δ 170.4, 169.8, 169.6, 169.5, 169.4, 168.7, 168.6, 168.4 (O*C*OMe **1**, **3**, **11-15**), 146.1 (C-3’ **11**), 146.0 (C-3’ **14**), 144.8 (C-4’ **15**), 144.7 (C-4’ **12**), 143.0 (C-3’ **1**), 142.9 (C-3’ **3**), 141.6 (C-4’ **1**), 141.5 (C-4’ **3**), 137.7 (C-3’ **12**), 136.9 (C-1’ **11**), 136.8 (C-1’ **15**), 136.3 (C-4’ **11**), 136.1 (C-4’ **14**), 130.6 (C-1’ **12**), 130.3 (C-1’ **1**), 129.7 (C-1’ **3**), 126.7 (C-6’ **12**), 126.5 (C-6’ **15**), 126.4 (C-5’ **15**), 123.1 (C-2’ **13**), 122.5 (C-5’, C-6’ **13**), 122.0 (C-2’ **12**), 121.9 (C-2’ **15**), 121.6 (C-5’ **11**), 121.5 (C-5’ **14**), 120.6 (C-6’ **11**), 120.3, 120.2 (C-6’ **1**, **3**), 117.4 (C-2’ **11**), 117.3 (C-2’ **14**), 117.0 (C-5’ **12**), 116.9 (C-6’ **15**), 115.2, 115.0 (C-2’ **1**, **3**), 114.6 (C-5’ **1**), 114.5 (C-5’ **3**), 64.3 (C-1 **3**), 63.9, 63.8 (C-1 **14**, **15**), 62.9 (C-1 **1**), 62.7 (C-1 **12**), 62.6 (C-1 **11**), 62.4 (C-1 **13**), 37.7 (C-2 **11**), 37.6, 37.5 (C-2 **15**, **1**), 37.3 (C-2 **12**), 33.7 (C-2 **14** o **15**), 33.6 (C-2 **3**), 33.3 (C-2 **14** o **15**), 28.8 (OCO*Me* **3**, **14**, **15**), 20.1, 20.0 (ArOCO*Me* **11-15**).

Spectroscopic data for crude reaction depicted in Scheme 5

^{1}H-NMR (700 MHz, $(CD_3)_2CO$): δ 7.12 (dd, 1H, *J*= 8.5 Hz, *J*= 2.3 Hz, H-6’ **20**), 7.08 (d, 1H, *J*= 2.3 Hz, H-2’ **20**), 7.07 (dd, 1H, *J*= 8.5 Hz, *J*= 2.3 Hz, H-6’ **17**), 7.06 (d, 1H, *J*= 2.3 Hz, H-2’ **16**), 7.04 (dd, 1H, *J*= 2.3 Hz, H-2’ **17**), 7.03 (d, 1H, *J*= 2.3 Hz, H-2’ **19**), 6.98 (d, 1H, *J*= 8.5 Hz, H-5’ **16**), 6.94 (2d, 1H, *J*= 8.5 Hz, H-5’ **19**, **20**), 6.91 (d, 1H, *J*= 8.5 Hz, H-5’ **17**), 6.90 (d, 1H, *J*= 2.3 Hz, H-2’ **18**), 6.88 (dd, 1H, *J*= 8.5 Hz, *J*= 2.3 Hz, H-6’ **19**), 6.87 (d, 1H, *J*= 2.3 Hz, H-2’ **4**), 6.84 (dd, 1H, *J*= 8.5 Hz, *J*= 2.3 Hz, H-6’ **16**), 6.78 (d, 1H, *J*= 8.5 Hz, H-5’ **18**), 6.75 (d, 1H, *J*= 8.5 Hz, H-5’ **4**), 6.72 (dd, 1H, *J*= 8.5 Hz, *J*= 2.3 Hz, H-6’ **18**), 6.68 (dd, 1H, *J*= 8.5 Hz, *J*= 2.3 Hz, H-6’ **4**), 4.85, 4.84 (2dd, 1H each, *J*= 7.5 Hz, *J*= 4.0 Hz, H-2 **19**, **20**), 4.75 (dd, 1H, *J*= 7.5 Hz, *J*= 4.6 Hz, H-2 **18**), 4.64, 4.63 (2dd, 1H each, *J*= 7.8 Hz, *J*= 4.2 Hz, H-2 **16**, **17**), 4.55 (dd, 1H, *J*= 8.2 Hz, *J*= 3.9 Hz, H-2 **4**), 4.16-4.04 (m, 6H, H-1 **18**, **19**, **20**), 3.59-3.46 (m, 6H, H-2

4, **16**, **17**), 2.25, 2.24, 2.23 (3s, 3H each, ArOCO*Me* **16**, **17**, **19**, **20**), 2.00, 1.99, 1.98 (3s, 3H each, OCO*Me* **18**, **19**, **20**).

^{13}C-NMR (175 MHz, $(CD_3)_2CO$): δ 171.0, 170.1, 170.0 (O*C*OMe **18-20**), 169.5, 169.4, 169.4 (ArO*C*OMe **16**, **17**, **19**, **20**), 149.3 (C-3' **16**), 149.5 (C-3' **19**), 149.3 (C-4' **20**), 148.9 (C-4' **17**), 146.0 (C-3' **18**), 145.9 (C-3' **4**), 145.7 (C-**18**), 145.3 (C-4' **4**), 142.3 (C-4' **16**), 141.3 (C-4' **19**), 139.4 (C-3' **17**), 139.3 (C-3' **20**), 138.9 (C-1' **16**), 138.6 (C-1' **19**), 135.1, 135.0 (C-1' **17**, **20**), 134.1 (C-1' **4**, **18**), 125.2, 125.1 (C-6' **17**, **20**), 123.6 (C-5' **16**), 123.4 (C-5' **19**), 121.9 (C-2' **20**), 121.8 (C-2' **17**), 118.4 (C-6' **18**), 118.3 (C-6' **4**), 118.1 (C-6' **16**, **19**), 117.5 (C-5' **20**), 117.3 (C-5' **17**), 116.0 (C-5' **18**), 115.8 (C-5' **4**), 115.7 (C-2' **16**), 115.6 (C-2' **19**), 114.5 (C-2' **18**, **4**), 75.1 (C-2 **4**), 74.9, 74.6 (C-2 **16**, **17**), 71.9 (C-2 **18**), 71.7, 71.4 (C-2 **19**, **20**), 70.1 (C-1 **18**), 69.8 (C-1 **19**, **20**), 69.1 (C-1 **4**), 68.9, 68.8 (C-1 **16**, **17**), 20.8, 20.7, 20.6, 20.5 (OCO*Me* **4**, **16-20**).

Spectroscopic data for crude reaction depicted in Scheme 6

^{1}H-NMR (500 MHz, $CDCl_3$): δ 7.13 (m, 4H, H-2', H-6' **22**, **26**), 7.08 (d, 1H, *J*= 7.4 Hz, H-5' **25**), 7.06 (d, 1H, *J*= 7.4 Hz, H-5' **21**), 7.01 (d, 1H, *J*= 1.9 Hz, H-2' **21**), 7.00 (d, 1H, *J*= 1.9 Hz, H-2' **25**), 6.98 (2d, 1H each, *J*= 7.4 Hz, H-5' **22**, **26**), 6.90 (2dd, 1H each, *J*= 7.4 Hz, *J*= 1.9 Hz, H-6' **21**, **25**), 6.89 (2d, 1H each, *J*= 1.9 Hz, H-2' **8**, **23**), 6.84 (2d, 1H each, *J*= 7.4 Hz, H-5' **8**, **23**), 6.80 (2dd, 1H each, *J*= 7.4 Hz, *J*= 1.9 Hz, H-6' **8**, **23**), 5.04 (s, 2H, H-1, **25**), 5.02 (2s, 2H, H-1, **26**), 4.98 (s, 2H, H-1 **23**), 4.75 (s, 2H, H-1 **24**), 4.69 (s, 2H, H-1, **8**), 4.63 (s, 2H, H-1 **21**), 4.61 (s, 2H, H-1 **22**), 2.36, 2.35 (2s, 3H each, ArOCO*Me* **21**, **22**, **24-26**), 2.08, 2.07 (2s, 3H each, OCO*Me* **23**, **25**, **26**).

^{13}C-NMR (125 MHz, $CDCl_3$): δ 170.8, 170.5, 170.3 (O*C*OMe **23-26**), 169.5, 169.4, 169.3 (ArO*C*OMe **21**, **22**, **24-26**), 147.2 (C-3' **21**), 147.2 (C-3' **25**), 146.8 (C-4' **26**), 146.6 (C-4' **22**), 144.1 (C-3' **23**), 143.8 (C-4' **23**), 140.2 (C-1' **23**), 138.7 (C-3' **22**), 138.0 (C-4' **21**), 134.1 (C-1' **21**), 128.9 (C-1' **22**), 127.5 (C-6' **26**), 125.9 (C-6' **22**), 123.0 (C-2' **26**), 122.7 (C-5' **21**, **25**), 121.6 (C-6' **23**), 121.5 (C-2' **22**), 120.9 (C-6' **25**), 119.4 (C-6' **21**), 117.9 (C-5' **22**, **26**), 117.7 (C-2' **25**), 116.3 (C-2' **21**), 116.0 (C-2' **23**), 115.4 (C-5' **23**), 66.3 (C-1 **23**), 65.7, 65.4 (C-1 **25**, **26**), 64.8 (C-1 **21**), 64.7 (C-1 **22**), 21.2 (OCO*Me* **23**, **25**, **26**), 21.1 (ArOCO*Me* **21**, **22**, **24-26**).
