# Supplementary material for: A Straightforward Access to New Families of Lipophilic Polyphenols by Using Lipolytic Bacteria
Source: PLoS One. 2016 Nov 17;11(11):e0166561. doi: 10.1371/journal.pone.0166561 (PMC5113952; doi:10.1371/journal.pone.0166561)
Supplement: S1 Table — (PDF) [file pone.0166561.s004.pdf]

S1 Table. **Selection of lipolytic microorganisms in the primary screening (hydrolytic)**

Sample	Bacteria	Fungi	Total
**HR11**	3	0	3
**HR12**	0	378	378
**HR21**	63	15	78
**Total**	66	393	459
