# Supplementary material for: A Straightforward Access to New Families of Lipophilic Polyphenols by Using Lipolytic Bacteria
Source: PLoS One. 2016 Nov 17;11(11):e0166561. doi: 10.1371/journal.pone.0166561 (PMC5113952; doi:10.1371/journal.pone.0166561)
Supplement: S2 Table — In red, the isolates selected with absorbance values higher than the highest hydrolysis control. (PDF) [file pone.0166561.s005.pdf]

S2 Table. **Absorbance values obtained in the transesterification assay. In red, the isolates selected with absorbance values higher than the highest hydrolysis control**

Isolate	Abs 410nm	Isolate	Abs 410nm	Isolate	Abs 410nm
HR21-1	0,229	HR21-23	0,271	HR21-45	0,001
HR21-2	0,102	HR21-24	0,109	HR21-46	0,023
HR21-3	0,289	HR21-25	0,024	HR21-47	0,067
HR21-4	0,065	HR21-26	0,145	HR21-48	-0,001
HR21-5	0,114	HR21-27	-0,001	HR21-49	0,023
HR21-6	0,233	HR21-28	0,275	HR21-50	0,018
HR21-7	0,096	HR21-29	0.292	HR21-51	0,038
HR21-8	0,095	HR21-30	0,404	HR21-52	0,152
HR21-9	0,088	HR21-31	0,062	HR21-53	-0,018
HR21-10	0,036	HR21-32	0,061	HR21-54	0,05
HR21-11	0,098	HR21-33	0,039	HR21-55	-0,062
HR21-12	0,14	HR21-34	0,084	HR21-56	-0,077
HR21-13	0,862	HR21-35	-0,001	HR21-57	0,023
HR21-14	0,069	HR21-36	-0,046	HR21-58	0,113
HR21-15	0,077	HR21-37	0,118	HR21-59	0,215
HR21-16	0,102	HR21-38	0,001	HR21-60	0,149
HR21-17	0,28	HR21-39	-0,07	HR21-61	0,114
HR21-18	0,258	HR21-40	0,093	HR21-62	0,17
HR21-19	0,087	HR21-41	0,065	HR21-63	0,184
HR21-20	0,627	HR21-42	-0,001	HR11-64	0,317
HR21-21	0,109	HR21-43	0,005	HR11-65	0,158
HR21-22	0,081	HR21-44	0,001	HR11-66	0,101
